# Super Infection of An Ovarian Dermoid Cyst with *Actinomyces* in An Infertile Woman

**Published:** 2013-07-31

**Authors:** Saghar Salehpour, Azadeh Akbari Sene

**Affiliations:** 1 Department of Obstetrics and Gynecology, Infertility and Reproductive Health Research Center , Shahid Beheshti University of Medical Sciences, Tehran, Iran; 2 Department of Obstetrics and Gynecology, Iran University of Medical Sciences, Shahid Akbar-abadi Hospital, Tehran, Iran

**Keywords:** Dermoid Cyst, Actinomycosis, Infection, Infertility, Intracytoplasmic Sperm Injection

## Abstract

We present super infection of an ovarian dermoid cyst with actinomyces in an infertile patient. This is a case-report study for evaluation a couple with male factor infertility, who was a good candidate for intracytoplasmic sperm injection (ICSI), while a 10 cm dermoid cyst was found in the woman’s right ovary. Patient complained of pelvic pain, intermittent fever, dysmenorrhea, and dyspareunia. The cyst was extracted using laparoscopy, whilst in histopathological examination, an actinomycosis super infection was reported. Actinomyc super infection of an ovarian dermoid cyst is a very rare incident which can also occur in women with no history of intrauterine device (IUD) usage or previous fertility.

## Introduction

Dermoid cyst (mature cystic teratoma) is a prevalent ovarian neoplasia which might become complicated by torsion, rupture, or dysplasia, whereas super infection is a rare complication of these cysts (1). Pelvic actinomycosis is also a rare infection in women, especially have been found in women with a long history of using intrauterine device (IUD) (2, 3). In this study, we present super infection of an ovarian dermoid cyst with actinomyces in an infertile patient.

## Case Report

An infertile couple of afghan origin with low socio-economic and educational state came to our center to treat their 10 years primary infertility. The primary cause of infertility was determined to be azoospermia of the 28 years old male partner, and the couple was a good candidate for surgical sperm retrieval (testicular sperm extraction/TESE) and intracytoplasmic sperm injection (ICSI).

In preliminary studies before ICSI, the 26 years old female partner reported regular menstruation intervals, but she experienced secondary dysmenorrhea and deep dyspareunia for about a year.

She also gave a history of unspecified manipulation of uterine cavity by a midwife in Afghanistan to treat her infertility before the onset of these findings. She also complained of intermittent pelvic pain and nocturnal fever without chills starting after this manipulation. In examination of pelvic area, a mobile cystic formation of about 10 cm in diameter was palpable in right adnexal region. Transvaginal ultrasound examination showed a cystic formation with a size of 101×42 mm, including hyper-echoic solid components and a mural nodule, in the right ovary, while color Doppler examination revealed a pulsatility index (PI) of higher than one, so both findings confirmed a benign ovarian teratoma.

In hysterosalpingography, uterine cavity was normal and both tubes were patent, but the evidence of an adnexal mass with three complete teeth was observed. All tumor markers were within the normal limits [carbohydrate antigen19-9 (CA19-9)=17.1, carbohydrate antigen 125 (CA125)=18.7, carcino embryonic antigen (CEA)=2.4, and alpha-1-fetoprotein (α FP)=0.2] and other laboratory findings were all normal, as well [white blood cells (WBCs)=6200, erythrocyte sedimentation rate (ESR)=2, C- reactive protein (CRP+1), purified protein derivative (PPD)=negative, and chest X-ray (CXR)=normal].

Patient underwent laparoscopy with a diagnosis of dermoid cyst. During laparoscopic exam pelvic cavity, uterus and left ovary had normal view, and both tubes were patent. Right ovary contained a big cyst with a 5×10 cm diameter without any adhesion to surrounding tissues.

**Fig 1:**
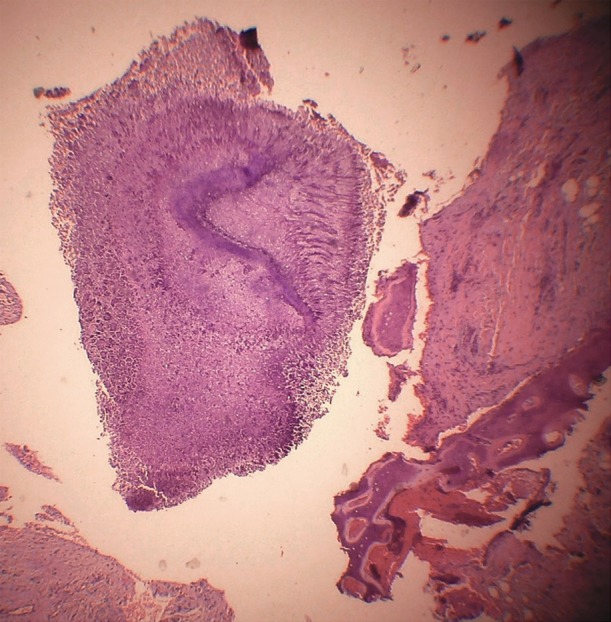
A Section of ovarian mature cystic teratoma showing fibro ossified tissue containing sulfur granule of actinomyces [using hematoxylin and eosin stain (H&E)].

**Fig 2:**
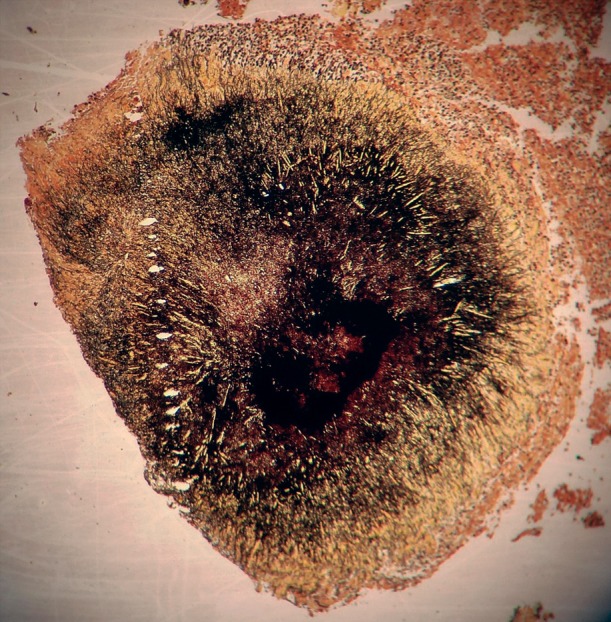
Methinamine silver staining of actinomycosis sulfur granule.

Grossly, the cyst contained abundant adipose tissue, osteoid material and four well-formed teeth. Laparoscopic cystectomy was performed, and the remaining ovarian tissue was preserved. The mass was sent for histopathologic evaluation. Microscopic sections showed ovarian cyst with ciliated stratified epithelium and foci of squamous lining. Components such as mature osseous and chondroid tissue were indentified. Some foci of acute and chronic inflammatory cells infiltration and actinomyces like organisms were also noted. The histopathologic findings suggested the presence of mature cystic teratoma with actinomycotic abscess formation (Fig 1). The diagnosis of actinomycosis was confirmed using silver methinamine staining (Fig 2).

Patient was treated for three months with amoxicillin 500 mg/TDS and doxycycline100 mg/BID after consultation with an infectious disease specialist. After the treatment period, patient’s fever and pelvic pain stopped. At this time, patient had serum follicle-stimulating hormone (FSH)=8.9 and serum anti-mullerian hormone (AMH)=1.4 and in ultrasonographic exam both ovaries had normal view.

ICSI was performed following a standard long protocol using gonadotropin-releasing hormone (GnRH) agonist test (Superfact, Buserelin, Aventis, Germany) and a combination of Fostimon and Merional (IBSA, Switzerland). Thirty six hours after injection of 10000 IU human chorionic gonadotropin (hCG), three oocytes were obtained from the left ovary. The right ovary remained inactive during the controlled ovarian hyperstimulation (COH) and no oocyte was retrieved from it. In total, two good quality embryos were formed and were transferred using transcervical technique. Serum βhCG level remained negative two weeks after embryo transfer.

## Discussion

Mature ovarian cystic teratoma or dermoid cyst is the most prevalent ovarian germ cell tumor comprising about 20% of all ovarian neoplasms. Dermoid cyst might become complicated by torsion, rupture or develop to a malignant tumor, but infection is a rare complication accruing in only 1% of mature ovarian cystic teratomas (1). A study by Hasanzadeh et al. have found that only seven cases of super infection of dermatoid cyst has been reported in the english literature, while the most common source of infection has been reported to be coliform bacteria. They also reported a case of anaerobic super infection of dermoid cyst and abscess formation in a 70-year-old patient (4).

Luk et al. reported a case of dermoid cyst infection following a dilation and curettage (D&C) procedure with signs of tubo-ovarian abscess (5). In rare cases, infection with *brucella* (6), salmonella (7) and *schistosomiasis* (8) have been reported. In a review of literature, we only found one case of actinomcosis super infection of dermoid cyst reported by Luckas et al., which is the second investigated case report in english literature (9). *Actinomyces* Spp. are gram positive, anaerobic, and nonspore-forming bacilli (10).

*Actinomyces* is a normal habitant of gastrointestinal tract and is also present in 5% of cervical smears from healthy women (2). IUD might facilitate the pelvic access by the microorganisem, (3) and in women using IUD, the chance of actinomycosis infection has been reported to be between 1.6 and 11.6% (11). Patients mostly report unspecified symptoms, like abdominal pain, weight loss, fever and foul-smelling vaginal discharge (12). Pelvic actinomycosis is a rare cause of pelvic masses, but should be considered particularly in women who have IUD in situ, or report a long history of IUD usage (13). In these cases, patient might present with malignant like symptoms, intestinal obstruction or acute peritonitis (14). Kumar et al. reported a 32 year-old woman with a history of previous IUD placement who presented with low grade fever, abdominal pain, weight loss, poor appetite for six months, and the presence of a 85×45 cm solid pelvic mass. The mass mimicked an invasive ovarian malignancy with following characteristic features: occupying both adnexae, involving mesocolic fat and sigmoid colon, and causing unilateral hydronephrosis. After an extensive laparotomy, the pathologic findings were suggestive for pelvic actinomycosis (12).

In a report of 11 cases of pelvic actinomycosis, Marret et al. (14) showed that all cases were related to long time IUD usage with an average of 8 years Seven patients in their series were diagnosed with acute peritonitis with or without bowel obstruction, and four had extensive surgical procedures for suspected ovarian cancer. In a review of related cases, we found that out of 58 reported cases, 52 had a long time history of IUD usage.

However, it should be considered that rare cases of pelvic actinomycosis without a history of IUD usage have been reported (10, 15-17). In a report by Munjal et al. (17) a 35-year-old woman with the history of infertility presented with fever, vaginal discharge and a 7×5.5 cm pelvic mass adherent to cecum. After panhysterectomy, the mass was diagnosed as the tubo-ovarian actinomycosis.

Our patient did not have any history of IUD usage or previous fertility. It is possible that manipulation of uterus in a non standard setting and using non sterile equipment in this patient had caused the transfer of germs into the uterus cavity and secondary infection of ovarian dermoid cyst.

Diagnosis of pelvic actinomycosis is hard, and the infection is mostly recognized during the surgery or in histopathological studies (12). Early detection and treatment with B-lactamase antibiotics, like our case, might negate the need for more extensive surgical procedures. An extended treatment with antibiotics for at least two months and in some cases up to one year should be considered to root out the infection (14). A good cooperation between the surgeon and an infectious disease specialist can result in better outcomes.

Our case shows that pelvic actinomycosis should be considered in women without a history of previous fertility or IUD usage even if the signs and symptoms are unspecific; especially the patient comes from a low level socio economic background. The presence of unusual findings like night fevers, chills, intermittent abdominal pain, and nonspecific digestive system signs can lead to consider a possible super infection of benign pelvic masses like dermoid cysts by the physician.

Our case and other cases reported in the literature indicate the importance of sending the samples of suspicious tissue during surgery for microbiological examination in parallel with cytopathologic examination, especially in the presence of these symptoms. Also, the long-term follow-up of patients after surgery has a high priority. There is a need for further studies on the outcome of COH/ ART in infertile women with pelvic actinomycosis.

## References

[1] Disaia PJ, Creasman WT, Disaia PJ, Ceasman WT (2002). Germ cell, stromal, and other ovarian tumors. Clinical gynecological.

[2] Valbø A, Rønning EJ, Aaberg M (2010). Actinomycosis as a complication of intrauterine device use. Tidsskr Nor Laegeforen.

[3] Burlando SC, Paz LA, De Feo LG, Benchetrit G, Rimoldi D, Predari SC (2001). Ovarian abscess due to Actinomyces sp.in absence of an intrauterine contraceptive device. Medicina (B Aires).

[4] Hasanzadeh M, Tabare SH, Mirzaean S (2010). Ovarian dermoid cyst. Professional Med J.

[5] Luk J, Quaas A, Garner E (2007). The superinfection of a dermoid cyst. Infect Dis Obstet Gynecol.

[6] Uwaydah M, Khalil A, Shamsuddine N, Matar F, Araj GF (1998). Brucella-infected ovarian dermoid cyst causing initial treatment failure in a patient with acute brucellosis. Infection.

[7] Matsubayashi T, Hamajima T, Asano K, Mizukami A, Seguchi M, Kohno C (2001). Salmonella infection of an ovarian dermoid cyst. Pediatr Int.

[8] Maleto M, Muuse MM, Hussein AM, Falconieri G (1987). Schistosomiasis in a cystic teratoma of the ovary. Clin Exp Obstet Gynecol.

[9] Luckas MJ, Cawdell GM (1995). Actinomycosis infection of a dermoid cyst mimicking pelvic malignancy. Aust N Z J Obstet Gynaecol.

[10] Lee YK, Bae JM, Park YJ, Park SY, Jung SY (2008). Pelvic actinomycosis with hydronephrosis and colon stricture simulating an advanced ovarian cancer. J Gynecol Oncol.

[11] Chatwani A, Amin-Hanjani S (1994). Incidence of actinomycosis associated with intrauterine devices. J Reprod Med.

[12] Kumar N, Das P, Kumar D, Kriplani A, Ray R (2010). Pelvic actinomycosis mimicking: an advanced ovarian cancer. Indian J Pathol Microbiol.

[13] Akhan SE, Dogan Y, Akhan S, Iyibozkurt AC, Topuz S, Yalcin O (2008). Pelvic actinomycosis mimicking ovarian malignancy: three cases. Eur J Gynaecol Oncol.

[14] Marret H, Wagner N, Ouldamer L, Jacquet A, Body G (2010). Pelvic actinomycosis: just think of it. Gynecol Obstet Fertil.

[15] Atay Y, Altintaş A, Tuncer I, Cennet A (2005). Ovarian actinomycosis mimicking malignancy. Eur J Gynaecol Oncol.

[16] Marwah S, Marwah N, Singh I, Singh S, Gupta A, Jaiswal TS (2005). Ovarian actinomycosis in absence of intrauterine contraceptive device: an unusual presentation. Acta Obstet Gynecol Scand.

[17] Munjal K, Nandedkar S, Subedar V, Jain S (2010). Tubo-ovarian actinomycosis mimicking as ovarian malignancy: report of three cases. Indian J Pathol Microbiol.

